# Seasonal Variations in Trace Metals and Anions in Surface Water of the Vaal River, South Africa: Implications for Human Health Risk Assessment

**DOI:** 10.1007/s00128-026-04202-5

**Published:** 2026-03-07

**Authors:** Fadzanai Fusirai, Luke Chimuka, Heidi Richards

**Affiliations:** School of Chemistry, Molecular Sciences Institute, University of the Witwatersrand, Johannesburg, South Africa

**Keywords:** Total recoverable metals, Water quality index (WQI), Carcinogenic risk, Non-carcinogenic risk

## Abstract

This study monitored seasonal variations of metals and anions in the Vaal River and assessed health risks from water use. Water samples were collected over four seasons from locations spanning about 180 km and analyzed for physical and chemical properties. Concentrations of Fe, Al, and Mn exceeded World Health Organization (WHO) and South African National Standards (SANS) limits. Electrical Conductivity (EC) exceeded standards in all seasons except autumn. One-way ANOVA indicated no significant differences over seasons except for Cu, while the Kruskal Wallis test showed significant variation in Br-, Cd, and Cr. Water Quality Index (WQI) ranged from 74.6 to 255.3, indicating water quality from good to very poor, with Al and Fe as major contributors to poor quality. Human health risk assessments revealed significant non-carcinogenic and carcinogenic risks for children at all sites, with Cr identified as the primary contributor to the estimated cancer risk.

## Introduction

Anions and trace metal contamination of surface waters have been a problem of great concern due to their threat to human and environmental health (Akoto et al. 2021). The pollution is not limited to natural processes, such as erosion; it also emanates from industrial activities like excessive use of fertilizers, mining activities, and disposal of sewage waste (Molekoa et al. 2021). Pollutant concentration in surface water also varies seasonally, due to the seasonal differences in rainfall and temperature. In aquatic environments, trace metals often sink into sediments, making them carriers of trace metals. Seasonal hydrological changes can cause sediment resuspension and shifts in water chemistry (e.g., pH, alkalinity, redox conditions), which affect trace metal mobility and bioavailability, resulting in their release into overlying surface water (Ccanccapa-Cartagena et al. 2023).

In South Africa, numerous dams feed from surface waters such as rivers, making them vital components of water supply. The Vaal River system is crucial for water resource services, supporting approximately 60% of the national economy and supplying water to over 45% of South Africa’s population, which is around 20 million individuals (Remilekun et al*.* 2021). Due to the limited availability of potable water in rural areas, several South Africans use river water for their basic needs (Edokpayi et al. 2018; Madikizela et al. 2023). Exposure to contaminants typically through ingestion and dermal contact may have detrimental effects on human health (da Silva Bonifácio et al. 2021). Even at low concentrations, pollutants in drinking water can cause long-term effects due to chronic exposure to these substances, like Cr and Cd, which are known to be cancer-causing metals (Sharma 2020).

Studies on seasonal trends of metals and anions concentrations in rivers across the world have been reported in the literature (Hossain et al. 2020; Guo et al. 2022; Ccanccapa-Cartagena et al. 2023). However, there are limited studies on the assessment of the Vaal River pollution, with metals monitored only at wastewater discharge points in the Vaal Basin (Moloi et al. 2020). Other studies have focused on microplastics in the Vaal River (Ramaremisa et al. 2022). Yet the Vaal River, which is South Africa’s second largest river and is referred to as South Africa’s workhorse, has been widely reported to be heavily polluted (Mnguni 2022). Therefore, this study hypothesized that seasonal changes significantly influence the concentrations of anions and total dissolved metals in the Vaal River, leading to spatial and seasonal variability in associated human health risks across different sampling sites.

## Materials and Methods

### Study Area

**Fig. 1:**
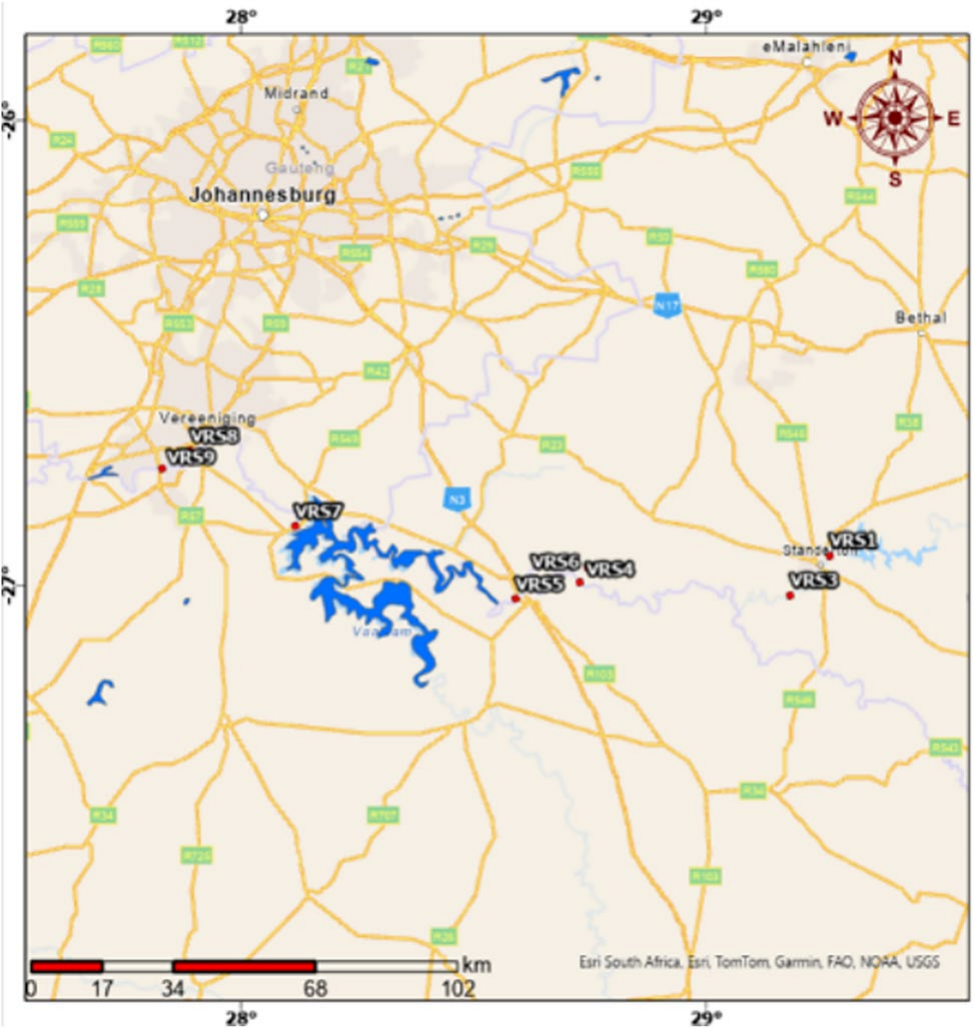
Map of the Vaal River sampling area from Standerton to Vanderbijlpark

The Vaal River flows year-round, with seasonal flow variations. The study area includes a 180 km stretch from Standerton to Vanderbijlpark, which features various activities such as mining and farming. Sampling sites, ranging from VRS1 (upstream) to VRS9 (downstream), were selected based on the proximity to industries, dams, towns, and the river’s topology (see Fig. 1).

### Sampling and Sample Analysis

Duplicate samples were collected in Autumn, Winter, Spring of 2023, and Summer of 2024. Total dissolved oxygen (TDS), electrical conductivity (EC), and pH were measured in the field using a portable Hanna Instrument multimeter. The procedure for water sample collection was followed as described by the USEPA (USEPA 1993). Samples were transported in cooler boxes to the lab, where metal samples were acidified to below pH 2 with nitric acid and stored at 4 ºC. Anion samples were filtered through 0.45 µm syringe filters and also stored at 4 ºC until analysis.

For total recoverable metal analysis, microwave-assisted acid digestion was employed using an Anton Paar Multiwave 5000 for extraction (Ojeda and Rojas 2019). To this end, a 12.5 mL water sample, 5 mL HNO_3_ and 2.5 mL HCl were placed in a polytetrafluoroethylene (PTFE) vessel. The mixture was digested with a ramp time of 10 min to 120 ºC and held for 15 min. After digestion, the 20 mL samples were transferred to plastic centrifuge tubes, filtered through 0.22 µm syringe filters, and analysed using ICP-OES (Spectro Genesis, Germany) and ICP-MS (NexION 2000, PerkinElmer, USA). Calibration was conducted using multi-element aqueous certified reference material (CRM) from De Bruyn Spectroscopic Solutions (Johannesburg, South Africa). For anion analysis, triplicate samples were analysed using an automated Metrohm Eco Ion Chromatography (Switzerland). Dilutions of a 100 mg/L single-element stock solution were used for the calibration (Edokpayi et al. 2022). The stationary phase was a Metro Supp 17–150/4.0 column. 5.0 mmol/L Na_2_CO_3_ and 0.1 mmol/L NaHCO_3_ eluents were used, 0.1 M H_3_PO_4_ solution was used as the suppressor, with the flow rate at 1 mL/min.

Analytical blanks and quality control standards were used to maintain quality (Safiur Rahman et al. 2021). Anions were above the limit of detection and ranged between 0.14 and 0.98 mg/L, with percentage recoveries of 90–106%. Similarly, metal concentrations determined using ICP-MS were above the limit of detection and ranged from 0.13 to 2.64 µg/L, with percentage recoveries of 82–102%. Metal concentrations determined using ICP-OES ranged between 0.15 and 0.67 mg/L, and percentage recoveries of 89–106%. The presence of analytes was distinguished from background noise with 99.7% confidence, thus, the instruments and methods used were accurate and reliable. The relative standard deviation (RSD) for all analytes was within acceptable limits of less than 5%.

### Water Quality Index (WQI) Evaluation

WQI reflects the combined impact of various parameters on water quality. Each parameter was assigned a weight (Wi) from 1 to 5 based on its importance in assessing drinking water quality (Gogoi et al. 2020). The relative weight (W_*i*_) computation formula is shown by Eq. (1) (Şener et al. 2017). The water quality rating scale was determined using Eq. (2).

1 $$ Wi = \frac{{w_{i} }}{{\mathop \sum \nolimits_{i = 1}^{n} w_{i} }} $$

2 $$ {\mathrm{q}}_{{\mathrm{i}}} = \frac{{C_{i} }}{{S_{i} }} \times 100 $$

Where W_i_ is the relative weight, *n* is the pollutant variables’ total number, *w*_*i*_ is each parameter’s weight, q_i_ is the quality rating, c_i_ is each chemical parameter’s concentration in each water sample in mg/L, and s_i_ is each chemical parameter’s drinking water standard in mg/L. The WQI was calculated using Eq. 3 and values were classified into 5 categories: < 50 for excellent water, 50–100 for good water, 100–200 for poor water, 200–300 for very poor water, and > 300 for unsuitable for drinking (Madilonga et al. 2021; Yakovlev et al. 2022). The water quality parameter with the greatest influence was identified using the effective weight (Ewi) (Eq. 4), where Sl_i_ is the subindex of the parameter (Eq. 5).

3 $$ {\mathrm{WQI}} = \sum\nolimits_{i = 1}^{n} {w_{i} } \times q_{i} $$

4 $$ {\mathrm{Ew}}_{{\mathrm{i}}} = \frac{{{\mathrm{SI}}_{{\mathrm{i}}} }}{{{\mathrm{WQI}}}} \times 100 $$

5 $$ {\mathrm{SI}}_{{\mathrm{i}}} {\text{ = W}}_{{\mathrm{i}}} {\kern 1pt} {\mathrm{x}}{\kern 1pt} {\kern 1pt} {\mathrm{q}}_{{\mathrm{i}}} $$

### Quantitative Health Risk Assessment

The human health risk assessment makes use of a mathematical model that allows the evaluation of the impact of trace metals on human health. The method gives an estimation of exposure dose and quantifies the risk to humans (Madilonga et al. 2021). The main pathway of elemental exposure, ingestion, was considered in this study (Kan et al. 2021). Equation 6 was used for Chronic Daily Intake (CDI) computation for both adults and children as proposed by the United States Environmental Protection Agency (USEPA). Assumptions were; average time (AT), children-3650 days, adults-25550 days; Body weight (BW), children-15 kg, adults-70 kg; exposure duration (ED), children-10 years, adults (70 years); ingestion rate (IR), children 1L/day, adults 2.2 L/day; exposure frequency (EF), 365 days per year for both children and adults.

6 $$ {\mathrm{CDI}}_{{{\mathrm{ingestion}}}} = \frac{Cwater \times IR \times EF \times ED}{{BW \times AT}} $$

where *Cwater* (mg/L) is the metal concentration in water.

The hazard quotient was computed using the Eq. (7).

7 $$ {\mathrm{HQ}} = \frac{CD1}{{RfD}} $$

The Reference Dose (RfD) values (mg/kg-day) for Cr, Cu, Fe, Zn, Mn, Ni, Cd, and Al are 0.003, 0.04, 0.7, 0.3, 0.024, 0.02, 0.001, and 1, respectively (Madilonga et al. 2021). The resulting HQ values indicate undesirable non-carcinogenic risk where HQ exceeds 1 or acceptable non-carcinogenic risk where HQ is less than 1. A summation of all HQs gave the potential non-carcinogenic risk associated with all metals, hazard index (HI) (Eq. 8). Where HI is greater than 1, this indicates non-carcinogenic health risks at the sampling site (Aswal et al. 2023).

8 $$ {\mathrm{HI}} = \sum {{\mathrm{HQ}}} $$

9 $$ {\mathrm{CR}} = {\mathrm{CDI}}{\mkern 1mu} {\mathrm{x}}{\mkern 1mu} {\mathrm{CSF}} $$

The potential Cancer Risk (CR) posed was determined by Eq. (9). Where CSF is the cancer slope factor. Metals referred to as carcinogens or cancer-causing metals in this study are Cd and Cr, with CSFs of 15 and 0.5 (mg/kg-day) respectively (Khan et al. 2021).

## Results and Discussion

### Physicochemical Parameters of the Vaal River

Total dissolved solids (TDS), Electrical Conductivity (EC), and pH readings from the Vaal River were compared to the SANS and WHO limits (Table 1).

**Table 1:** Seasonal ranges of physicochemical parameters in the Vaal River surface water compared with SANS and WHO guideline limits

	pH	EC (µS/cm)	TDS (mg/L)
SANS	≥ 5–≤ 9.7	≤ 170	≤ 1200
WHO	6.5–8.5	600	–
Summer	7.4–8.4	141.8–828.6	107–398
Autumn	7.4–8.4	145.1–466.9	90–233
Winter	7.5–8.5	177.6–794.4	88–346
Spring	7.3–8.5	174.4–845.3	92–462

The pH values ranged from 7.3 to 8.5 at different sampling sites over the seasons, which fell within the acceptable drinking water limits for both WHO and SANS. The average pH value was 7.93, indicating an alkaline nature of the river, attributable to the soil and rock–water interactions as the river flows (Zhang et al. 2018) and possible industrial and sewage effluents discharged into the river (Hassan et al. 2023). EC varied from 141.8–845.3 µS/cm, exceeding the maximum permissible limits over all seasons except in autumn. The average EC value was 409.4 µS/cm while TDS ranged from 90 to 462 mg/Land averaged to 227 mg/L, depicting a moderate level of ionic activity in the study area. Both parameters indicate some degree of nutrient loading or contamination from sewage water, which in the case of alkaline water, indicates concentrations of calcium and magnesium bicarbonates (Zhu et al. 2021).

### Anion Concentration in the Vaal River

Most anion concentrations were within WHO and SANS limits, except nitrite and nitrate which exceeded SANS limits (Table 2). There was high nitrite (1.54 mg/L) and nitrate (14.92 mg/L) at VRS3 and VRS8 during winter and summer, respectively. This could be due excessive use of nitrate fertilizers used for agricultural activities at that time (Zhu et al. 2021). Exposure to nitrate-contaminated water poses health risks to humans and may cause methemoglobinemia in infants (Zhang et al. 2018). The Shapiro–Wilk Normality Test satisfied the requirements for a non-parametric Kruskal–Wallis test for anions. The Kruskal–Wallis test revealed there was no statistically significant difference (*p* > 0.05) in seasonal concentrations except for Br- (*p* < 0.05).

**Table 2:** Ranges of anion concentrations in the Vaal River surface water compared with SANS and WHO guideline limits

Anion	Vaal River (mg/L)	SANS limit(mg/L)	WHO limit (mg/L)
Chloride	7.2–69.7	300	–
Fluoride	ND–0.4	1.5	1.5
Bromide	ND–1.3	–	–
Nitrite	ND–1.5	0.9	3
Nitrate	1.0–14.9	11	50
Sulphate	13.3–248.3	250	–
Phosphate	ND–3.5	–	–

–  Not established

### Metal Concentrations in the Vaal River

Metal concentrations were within the acceptable WHO and SANS drinking water limits except Al, Mn, and Fe for all seasons and Cr in autumn (Table 3). Al is moderately toxic except at high concentrations. Highest Al concentrations were recorded at VRS3 (13.73 mg/L) and VRS5 (12.99 mg/L) in autumn and summer, respectively, and a similar trend was observed for Fe. This could be attributed to the high surface runoff in wet seasons and a relatively high iron abundance on Earth (Edokpayi et al. 2016). Mn was in high concentration at downstream sampling sites; the same trend was also observed for Ca, Mg, Na, and K. Cu, Cr, Ni, and Zn showed similar trends over the seasons, with high concentrations at VRS3 in autumn, where Cr exceeded the maximum drinking water limit (> 0.05 mg/L). Cr is carcinogenic and is associated with lung cancer (Agbasi et al. 2023)**.** Metal concentrations satisfied the requirement for either parametric (One-way ANOVA) or non-parametric tests (Kruskal–Wallis). The Kruskal–Wallis test (*p* > 0.05) showed no statistically significant differences for Al, Ca, and Fe except for Cd and Cr (*p* < 0.05). One-way ANOVA showed that Na, K, Mg, Mn, and Ni concentrations did not vary significantly (*p* > 0.05) except for Cu (*p* < 0.05).

**Table 3:** Ranges of metal concentrations in the Vaal River surface water compared with SANS and WHO guideline limits

Metal	Vaal River (mg/L)	WHO (mg/L)	SANS (mg/L)
Cr	0.001–0.093	0.05	0.05
Cu	0.001–0.016	2.00	2.00
Fe	0.294–14.862	0.3	0.2
Zn	0.48–1.67	3.00	5.00
Mn	0.006–1.173	0.40	0.40
Al	0.411–13.728	0.20	–
Ni	0.010–0.067	0.07	0.07
Cd	ND–0.0003	0.003	0.003
Ca	13.33–33.76	300	–
Mg	7.949–27.81	–	–
K	2.106–10.24	–	–
Na	7.078–60.798	200	–

–  Not established

### Assessment of Water Quality Using WQI

**Fig. 2:**
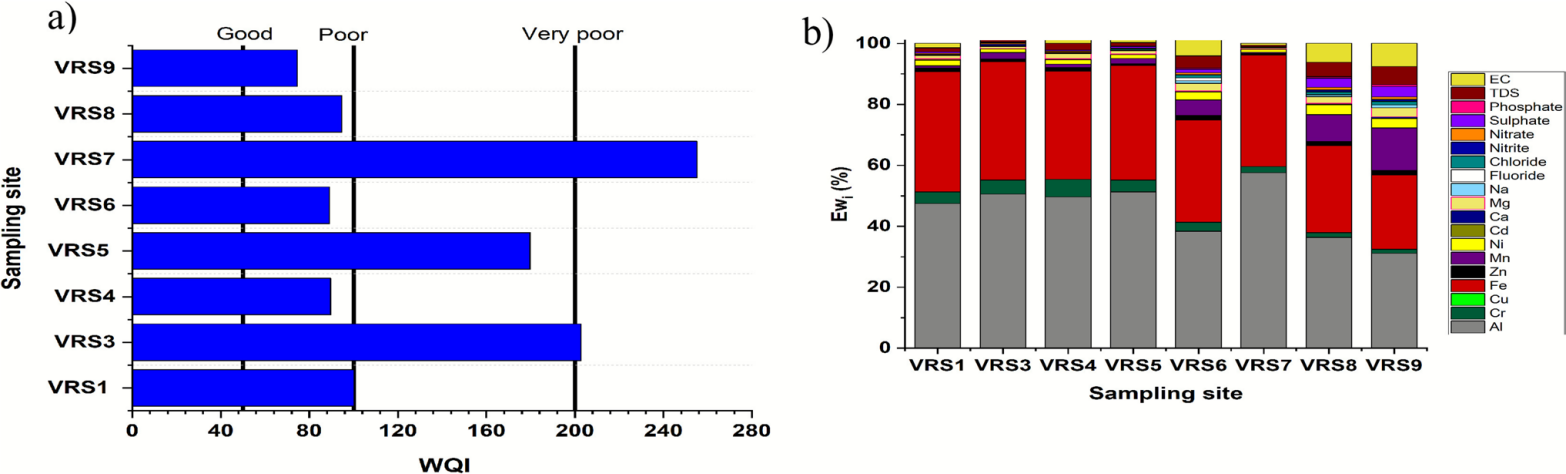
**a** Average WQI per sampling site and **b** Average Ew_i_ for each parameter per sampling site

The Vaal River water quality assessment for drinking water purposes was done through the evaluation of WQ1 (Rupias et al. 2021). Average concentrations for all analytes from all seasons were considered for WQI calculations. The WHO set limits for drinking water were used as references for quality index (qi) calculations. Each parameter was assigned a weight according to its importance in drinking water (Hammoumi et al. 2024). Cd was assigned a weight of 5 due to its toxicity and effect on drinking water quality and human health, while parameters like Ca were assigned a weight of 2, as it is less toxic to humans. Water quality values were calculated for each sampling site (Fig. 2a).

The calculated WQI values were in the range of 74.6–255.3, indicating that the quality of water along the catchment area varied from good to very poor water. Fe and Al were the main contributors to WQI at all sampling sites (Fig. 2b). VRS4, 6, 8, and 9 fell between the good and poor water categories, although most analytes showed an increase in concentration downstream. This is because Ca, Na, and other analytes that showed an increase downstream are assigned lower weights (w_i_), while Cr, Cu, and other analytes with more weight were in lower concentrations at these sampling sites. VRS1, 3, 5, and 7 fell between poor and very poor water quality. The most toxic metals were in high concentrations at VRS3 and VRS7, thus resulting in very poor water quality at these sampling sites.

### Principal Component Analysis (PCA)

**Fig. 3:**
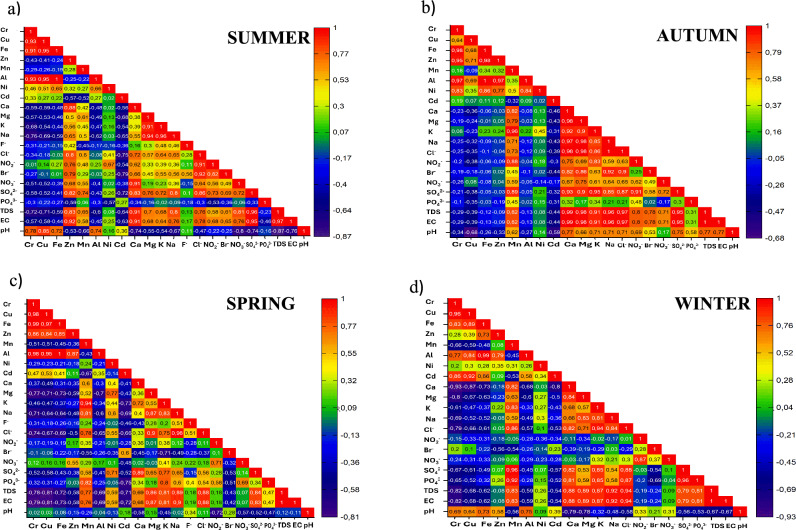
Pearson correlation coefficient matrix for **a** summer **b** autumn **c** spring **d** winter

To understand how analysed parameters interrelate, Pearson’s correlation matrix was prepared for each season and Fig. 3a–d shows the heatmaps of correlation coefficients. The results show a strong positive correlation between EC and TDS (r ≥ 0.97), indicating a directly proportional relationship across all seasons. Typically, EC increases as more solids are dissolved in water (Madilonga et al. 2021), hence the observed relation. The strong correlations between both EC and TDS with Mn, Cl^−^, SO_4_^2−^, Ca, Mg, K, and Na indicate that these parameters contributed significantly to water mineralization in the river, these metals and inorganic compounds also form complexes with each other (Varol, 2020; Gogoi, Taki and Kumar, 2020b). Cu, Cr, Al, and Fe did not contribute to water mineralization as they showed negative correlations with EC and TDS. However, they had strong positive correlations with each other, suggesting that they have common sources in the river (Hasan et al. 2021).

**Fig. 4:**
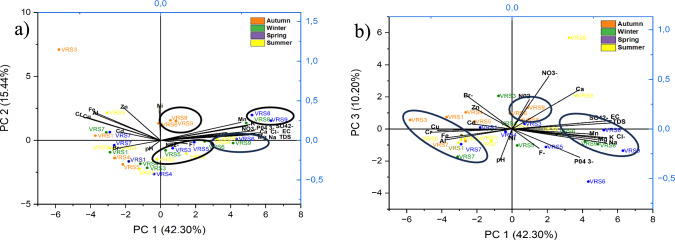
PCA scores plots **a** PC1 versus PC2 **b** PC1 versus PC3

To further compress the dimensionality of the dataset with correlated variables, while retaining its variation, PCA was used. Six principal components (PCs) with eigenvalues > 1 were extracted explaining 84.88% of the total variance. From these results, components of PC1 mainly constitute Mn, Mg, K, Na, Cl−, SO_4_^2−^, PO_4_^3−^, TDS and EC. Components of PC2 mainly consist of Cr, Cu, Zn, Al, Fe, Ni, and Cd. Components of PC3 mainly consist of Ca and NO_3_^−^. Other variables indicated a smaller but non-negligible weight in other principal components. PCA scores aligned each sampling site to a principal component. Clustering together of sites in a scores plot indicates similar pollution profiles. PC1, as the primary pollution source, representing the largest amount of variation, was plotted against PC2 and PC3 in Fig. 4. PC2 identifies secondary pollution, while the PC3 represents the minor pollution sources. PC4, PC5, and PC6 usually incorporate rare pollution sources or residual variability.

### Pollution Source Identification

Variables in PC1 represent the mineral component of the river or its geology, and pollutants could mainly be a result of carbonate minerals dissolution (Varol 2020; Zhang et al. 2021). Downstream sampling sites, VRS6, VRS8, and VRS9 are the primary sites exhibiting high pollution with high positive scores in PC1. Variables in PC2 represent metal pollution that mainly originates from anthropogenic activities, including mining, industrial activities, and wastewater contamination. VRS3 and VRS7 have high positive scores in PC2, making these sampling sites the major pollution contributors of metals. VRS3 is situated near industrial areas, including metal scrap yards and a coal supplier, as well as a non-functional wastewater treatment plant that discharges untreated wastewater into the river. VRS7 is close to the Vaal Dam outflow, where water from various areas accumulates, leading to metal deposits in the river. Variables in PC4 represent possible localised pollution, due to the high loading of NO_2_^−^. NO_2_^−^ is often linked to contamination from sewage and wastewater discharges (Balejčíková et al. 2020). PC5 and PC6 variables are the rare pollutants with F^−^ having a strong loading in PC6. VRS6 had high scores in PC6, indicating it could be the main site polluted with F^−^.

### Human Health Risk Assessment

**Fig. 5:**
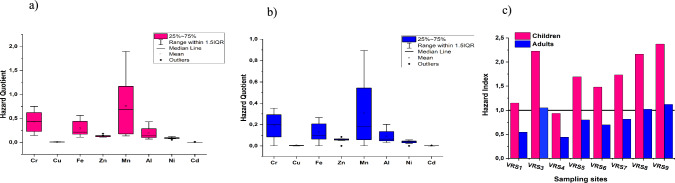
Box and Whisker plots showing Hazard Quotient in **a** Children **b** Adults and **c** Hazard Index values per sampling site for both children and adults

Potential non-carcinogenic risks were estimated, the range for HQ values is shown in the box and whisker plots in Fig. 5a, b. The HQ range for all sampling sites in children and adults was less than 1 for all metals, except for Mn in children at some sampling sites. The HQ range for Mn varied between 1.334 and 1.897, which indicates a risk. Consumption of Mn in high concentrations promotes the risk of temporary amnesia in children (Edokpayi et al. 2016). The HI values for children exceed 1 at all sites except VRS4, while HI values for adults only slightly exceeded 1 at VRS3 and VRS7, reflecting potential human health risks (Fig. 5c). The increasing order of human health risks can be presented as VRS4 < VRS1 < VRS6 < VRS 5 < VS7 < VRS3 < VRS8 < VRS9.

**Fig. 6:**
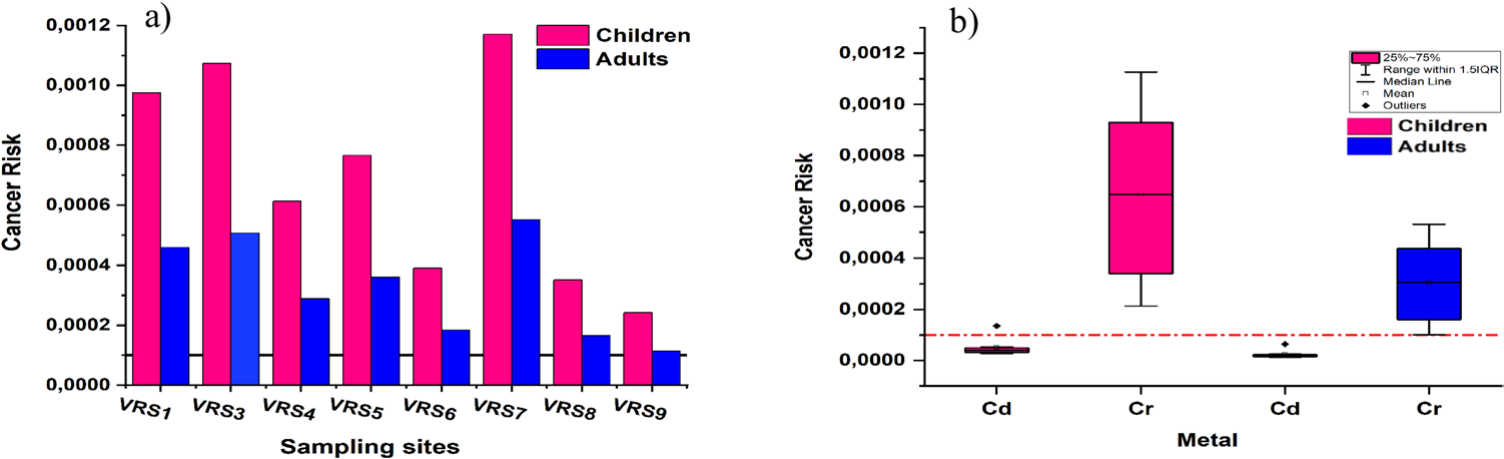
The **a** Cancer risk per sampling site for children and adults **b** Box and Whisker plots showing Cd and Cr contribution to CR in both children and adults

The mean cancer risk (CR) was 1.64 × 10^−4^ for adults and 3.49 × 10^−4^ for children, indicating that both groups face potential cancer-related health issues, with children at higher risk. VRS7 posed the greatest risk among sampling sites, while VRS9 had the least. The CR ranking from lowest to highest is VRS9 < VRS8 < VRS6 < VRS4 < VRS5 < VRS1 < VRS3 < VRS7 (Fig. 6a). Cd recorded CR values below the limit (1 × 10^−4^) at all sites in adults and children, except for VRS1 in children, where it reached 1.35 × 10^−4^, indicating a slight risk. In contrast, Cr consistently exceeded the 1 × 10^−4^ threshold at all sites for both groups, posing a high risk (Fig. 6b). Cr and Cd contributed 92.7% and 7.3% to cancer risk, respectively, indicating that of the analysed metals, Cr is the most harmful metal in the Vaal River catchment area. Children face higher carcinogenic and non-carcinogenic risks than adults, as they consume more food and water relative to their body weight, increasing their vulnerability to health hazards (Li et al*.* 2021). Children’s developing immune and nervous systems also make them more vulnerable to health risks from metals.

## Conclusion

This study analysed the seasonal variation of twenty-two water quality parameters. Statistical analyses revealed an insignificant impact of seasonal changes on the concentration of most pollutants except for Cu, Cr, Cd, and Br^−^. WQI indicate a variation in the quality of the water along the catchment area from good to very poor water, with Al and Fe being the major contributors. The human health risk assessment revealed that children had serious non-carcinogenic health risks, especially around VRS8 and VRS9. Both children and adults are at risk of carcinogenic health risks, with Cr being the main carcinogen, specifically at VRS3 and VRS7. Although most of the metals’ concentrations were within the WHO standard limits, they still present risk of health effects under chronic exposure conditions. It may, therefore, be recommended that the sampling stations be afforded remedial measures to control contamination by anthropogenic activities in a bid to protect human health.
